# The effectiveness of interventions to improve the seasonal influenza vaccination uptake among nurses: A systematic review

**DOI:** 10.1177/17571774231208115

**Published:** 2023-10-20

**Authors:** Paula Flanagan, Maura Dowling, Duygu Sezgin, Jolita Mereckiene, Louise Murphy, Martina Giltenane, Peter Carr, Georgina Gethin

**Affiliations:** 1 School of Nursing, Psychotherapy and Community Health, 8818 Dublin City University, Dublin, Ireland; 2 School of Nursing and Midwifery, 8799 University of Galway, Galway, Ireland; 3 11388 Health Protection Surveillance Centre, Dublin, Ireland; 4 School of Nursing and Midwifery, 8808 University of Limerick, Galway, Ireland

**Keywords:** Seasonal influenza vaccination, immunisation programmes, nurses, vaccination, healthcare workers, seasonal influenza vaccination behaviours

## Abstract

**Background:**

Seasonal influenza is a significant cause of mortality and morbidity worldwide. Despite annual recommendations, influenza vaccination uptake rates are disproportionately lower among nurses compared to other health care professionals, especially when compared to physicians. Nurses have an additional risk of exposure to influenza infection due to the nature of their work.

**Aim:**

To determine the effectiveness of interventions in increasing seasonal influenza vaccination uptake among nurses

**Methods:**

Evidence on the effectiveness of interventions to improve seasonal influenza vaccination uptake among nurses was systematically reviewed. A comprehensive search of six electronic databases and grey literature was undertaken. A minimum of two reviewers completed study selection, data extraction and risk of bias assessment independently.

**Results:**

One hundred and thirty-four studies were identified of which one cluster randomised trial met the inclusion criteria. The results of the included study found the implementation of an intervention with multiple components increased nurses’ seasonal influenza vaccination rates during a single influenza season in geriatric healthcare settings in France. As the evidence in this review was very limited, it was not possible to make recommendations regarding which interventions were effective at increasing the seasonal influenza vaccination rate for nurses.

**Conclusion:**

This systematic review highlights a lack of high-quality studies that assessed interventions to improve the seasonal influenza vaccination of nurses. In view of the likelihood of influenza and the coronavirus (COVID-19) pandemic occurring together, it is imperative to have evidence on effective interventions for the nursing workforce and for policy decision makers.

## Introduction

Seasonal influenza is a key public health priority. Globally, annual epidemics contribute to a significant increase in morbidity and mortality among high risk groups (World Health Organization, 2018) with the majority of deaths occurring in those aged over 65 years (Nair et al., 2012). Contributing to this disease burden is the fact that influenza infection can be transmitted within healthcare settings between vulnerable patients, staff and visitors (Weber et al., 2010).

Healthcare workers (HCWs) are three times more likely to be infected with influenza compared to other adults working outside the health service (Kuster et al., 2011). Nurses have an additional risk of exposure to influenza infection compared to other HCWs due to the nature and duration of their work (Chen et al., 2010; Kuster et al., 2011). Nurses may not only acquire but also transmit and spread infection to vulnerable patients (Haviari et al., 2015). These vulnerable patients have a high case fatality rate and are also at higher risk of acquiring nosocomial influenza infections (Salgado et al., 2002). It is estimated that 25% of all HCWs are infected with influenza each year (Odelin et al., 1993) and the rate of asymptomatic infections in fact might be higher (Kuster et al., 2011). Evidence also suggests that HCWs who are asymptomatic or have mild influenza illness often continue to work (Weingarten et al., 1989) contributing to influenza transmission and nosocomial outbreaks within healthcare settings (Bridges et al., 2003).

Although a number of measures are available to prevent transmission, annual vaccination remains the cornerstone for influenza prevention and recommendations internationally highly favour vaccination for high risk individuals including HCWs (European Council, 2009; Centres for Disease Prevention and Control (CDC), 2015; Immunisation Guidelines for Ireland, 2013; World Health Organization, 2012). Although vaccine effectiveness can vary each year (Osterholm et al., 2012), the seasonal influenza vaccine is safe and effective (Michiels et al., 2011) with vaccine effectiveness reported to be 50%–90% in adults of working age (Michiels et al., 2011). Although the influenza vaccine has been criticized for this varying efficacy, it remains the primary method of preventing morbidity and mortality associated with influenza (Pitts et al., 2014).

Outbreaks of influenza in both hospitals and long-term care facilities (LTCFs) have been associated with low vaccination coverage among HCWs (Cunney et al., 2000; Osterholm et al., 2012; Salgado et al., 2002, 2004; Sayers et al., 2013). Even though nurses are known to be at higher risk of influenza infection (Chen et al., 2010; Kuster et al., 2011), they have one of the lowest vaccination uptake rates compared to all other HCWs (Bautista et al., 2006; Christini et al., 2007; De Juanes et al., 2007; Esposito et al., 2007; Leitmeyer et al., 2006; Martinello et al., 2003; O’Lorcain et al., 2019; Riphagen-Dalhuisen et al., 2012; Sartor et al., 2004; Wicker et al., 2010) with uptake rates much lower than national and international targets of 75% (European Council, 2009; Health Service Executive (HSE), 2018; World Health Organization, 2003).

Vaccination uptake rates and decision-making reasons for accepting or rejecting the influenza vaccine covers a wide diverse spectrum and in general are themed around attitudes, beliefs, level of knowledge and perceived risk associated with influenza and the vaccine (Hofmann et al., 2006; Hollmeyer et al., 2013; Riphagen-Dalhuisen et al., 2012). These factors have been found to vary according to individual categories of HCWs (Bouadma et al., 2012; Dominguez et al., 2014; Edelstein and Pebody, 2014; Livni et al., 2008; Martinello et al., 2003). For instance, good medical knowledge about influenza and the vaccine has been found to be associated with an increase in HCWs’ vaccine uptake (Dominguez et al., 2014; Flanagan et al., 2020; Livni et al., 2008; Martinello et al., 2003; Smith et al., 2016; Zhang et al., 2012), however, lower levels of knowledge about influenza has been found to negatively impact nurses’ vaccination uptake when compared to physicians (Dominguez et al., 2014; Livni et al., 2008; Martinello et al., 2003). In contrast, vaccination decisions have been found to be associated with professionalism (being a role model or for patient protection) among physicians (Bouadma et al., 2012). Therefore, due to the dissimilarities in vaccination decision-making among the different categories of HCWs, encouraging influenza vaccination is a complex issue.

The effectiveness of interventions has also been found to vary among different categories of HCWs, in particular interventions aimed at promoting vaccine uptake have been reported to be less successful among nurses and other health professionals when compared to physicians (Bouadma et al., 2012; Friedl et al., 2012). These findings suggest that different approaches to influenza vaccination are necessary among nurses and also other HCWs (Bouadma et al., 2012; Dominguez et al., 2014). Nurses are the largest workforce in the health service and identifying interventions that are effective in increasing seasonal influenza vaccine coverage among them is essential. Although there have been a number of systematic reviews conducted which focused on interventions among HCWs (Hollmeyer et al., 2013; Lam et al., 2010; Lytras et al., 2016; Rashid et al., 2016), they did not compare and evaluate the interventions implemented by each occupational category. To address this gap, the aim of this systematic review is to determine what interventions are effective in increasing the seasonal influenza vaccine uptake among nurses.

## Methods

**Figure 1.**
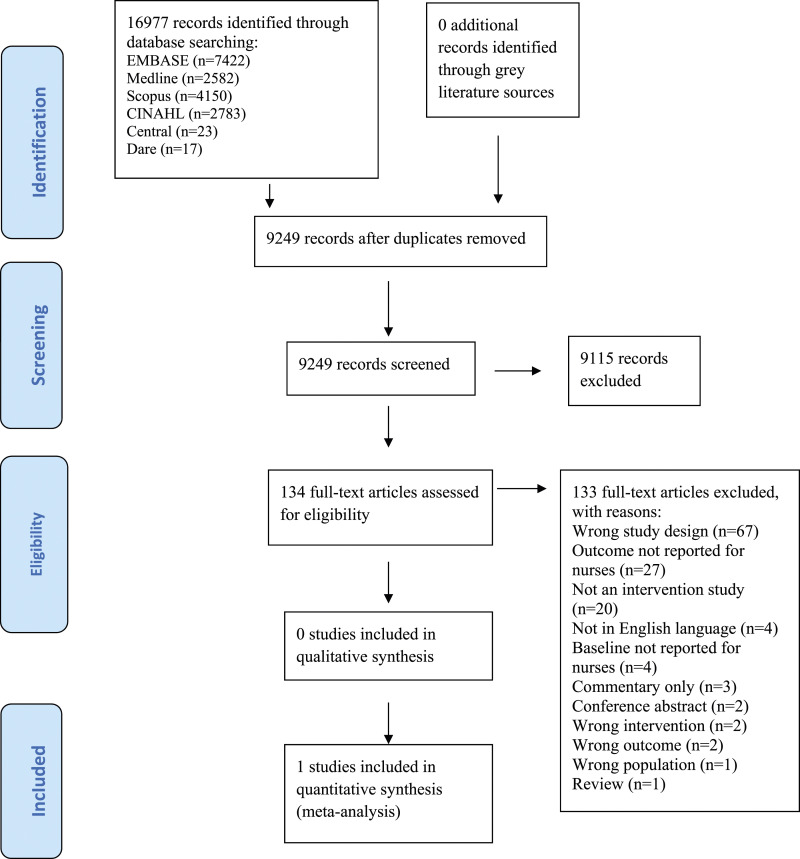
The ‘Preferred Reporting Item for Systematic Reviews and Meta-analyses’ (PRISMA) flow diagram for the selection and inclusion of studies (Moher et al., 2009).

This review followed a detailed protocol which is registered with Prospero (Flanagan et al., 2020). The Population, Intervention, Comparison and Outcome (PICO) model was used to formulate the research question for this review and is displayed in Table 1. The Effective Practice and Organisation of Care (EPOC) taxonomy of health systems interventions was followed and directed this systematic review (Cochrane, 2015). Identification of all relevant studies followed the preferred reporting item for a systematic review and meta-analysis (PRISMA) (Moher et al., 2009) (Figure 1).

**Table 1.** PICO framework.

PICO	Criteria
Population	Qualified nurses working in any healthcare setting
Intervention	Any intervention or strategy implemented with the aim of increasing seasonal influenza vaccination uptake rates among nurses. The US health care advisory committee on immunisation practices [50] recommend five components to improve HCWs immunisation rates. These include:
	• Educational interventions
	• Access to the vaccine
	• Staff incentives
	• Champions and role models
	• Leadership and management
	• Monitoring and feedback incorporated in single or multifaceted interventions
	For the purpose of this systematic review the intervention may consist of either single or multiple components.
Comparison	To another intervention or no intervention
Outcome	Primary outcome: Any change in seasonal influenza vaccine uptake among nurses
	Secondary outcome: None

### Search strategy

A comprehensive search of electronic databases and grey literature was undertaken. The following databases were systematically searched; MEDLINE Ovid (1946 to June 2020), EMBASE Ovid (1974 to May 2020), Cinahl (EBSCO Host, 1937 to June 2020) and Scopus. The Database of Abstracts of Reviews of Effects (DARE), Cochrane Database of Systematic Reviews and Cochrane Central Registry of Controlled Trials (CENTRAL) were also searched. The initial search was performed in Medline using a combination of controlled vocabulary and free-text terms to ensure maximum retrieval. The search terms were then adapted for EMBASE, Cinahl, Scopus, DARE and CENTRAL. No limits were applied to publication date. The initial search was not limited to English language only; however, this criterion was applied at full text screening stage. In order to identify further studies of interest, reference lists of all relevant studies and systematic reviews were tracked. Authors of trials were contacted for additional information where relevant. The following websites were also searched for grey literature: World Health Organization (WHO), European Centre for Disease Prevention and Control (ECDC), Centre for Disease Prevention and Control (CDC) and Public Health England (PHE). The results of the search strategy are displayed in the PRISMA flow chart in Figure 1 and the full search strategy is available in Appendix A.

### Eligibility criteria

Research studies that reported an intervention with the aim of increasing the seasonal influenza vaccination uptake among nurses were included. All eligible studies had to report the proportion or number of nurses who received the seasonal influenza vaccination as an outcome measure.

Randomised trials, non-randomised trials, controlled before and after studies, interrupted time series studies and repeated measure studies were identified as eligible for inclusion (Cochrane, 2015). Studies that reported all other vaccines including studies relating to a pandemic vaccine were excluded.

### Study selection

Records identified in the search were imported into Covidence software for systematic reviews and duplicates were removed. All results were screened independently by title and abstract and then by full text by at least two reviewers. Any conflicts were resolved by discussion.

### Data extraction and analysis

Two reviewers extracted the data of the included study using a modified version of the EPOC data extraction template (Cochrane, 2017). A narrative summary of the findings are presented and also displayed in Table 2.

**Table 2.** Data extraction summary table.

Author	Year	Design	Setting/Country	Year of study	HCW (*n*)	Nurses (*n*)	Intervention	Baseline vaccine rate of nurses^a^	Vaccination status of nurses	Intervention	Control	Total*
Rothan-Tondeur et al. (program 2)	2011	Cluster randomised controlled trial	Geriatric healthcare settings (*n* = 43) in France	2006–2007	1335 in the intervention group and 1539 in the control group	686 nurses; 347 in the intervention group and 339 in the control group	Program 2: Vesta study: incentive, slide show, two booklets, information leaflets, rubber bracelets and posters	33% in the intervention group and 36% in the control group	Vaccinated	170 (49%)	92 (27%)	262
									Unvaccinated	177 (51%)	247 (73%)	424
									Total	347	339	686

^a^Baseline vaccination uptake rate for nurses was 33% and 36% for the intervention and control group, respectively (i.e. after program 1 and before program 2).

*p value <.01.

### Methodology quality

The same two reviewers independently assessed the methodological quality of the included study using the EPOC risk of bias criteria (Cochrane, 2015). The risk of bias was assessed using the six domains of trial methodology: random sequence generation; allocation concealment; blinding of participants; incomplete outcome; and selective reporting and other bias. These six domains were assessed and classified as having high, low or unclear risk of bias (Table 3). The same two reviewers used the modified data collection form to independently extract the data from the included study (Table 2).

**Table 3.** Risk of bias assessment.

Cluster randomised controlled trial	Judgement	Support for judgement
Random sequence generation (selection bias)	Unclear	Insufficient information provided
Allocation concealment (selection bias)	Unclear	Insufficient information provided
Incomplete outcome data (attrition bias)	High	There were healthcare facilities lost to follow up but they had new facilities included in the control group between program 1 and program 2. Characteristics of those lost to follow up were not reported.
Selective reporting data (reporting bias)	Low	Not observed
Blinding of participants and personnel	High	Not observed
Blinding of outcome assessment (detection bias)	High	Not observed
Other bias		Recruitment bias: only HCWs agreeing to participate were included in the study and no information was collected on HCWs not included in the study. Therefore, a recruitment bias cannot be excluded. The 7 centres included in the control cluster between the implementation of the two active programs probably differed from the other centres that had immediately agreed to participate in the study. Crossover bias: Program 2 was developed and applied after Program 1, and potentially a small part of the effect of Program 2 may have been due to Program 1.

## Results

The initial search strategy yielded 16, 997 citations. No records were identified through searching the grey literature. After duplicates were removed (*n* = 7, 728), a total of 9, 249 records were screened by title and abstract (Figure 1). After closely examining the title and abstracts, a total of 134 articles were identified as potentially eligible and assessed by full-text. Only one study was identified as eligible and therefore included in this review.

### Characteristics of the included study

Rothan-Tondeur et al. (2011) reported a cluster randomised trial. The trial participants consisted of all HCWs employed in 43 geriatric health care settings in France (*n* = 1335 intervention group vs *n* = 1539 in the control group). This study formed program 2 of the VESTA study. The VESTA study was a cluster randomised interventional study and included two programs (program 1 and program 2) that were consecutively developed and assessed. The included study in this systematic review consists of the findings reported only from program 2 as baseline vaccination uptake rates for nurses was not reported for program 1. Program 1 was implemented between October 1^st^ and November 15^th^ 2005 (single component intervention study) and program 2 (multiple component intervention study) was implemented over one influenza season, between November 1^st^ and December 5^th^ 2006 (1 year after program one). The geriatric healthcare facilities that agreed to participate in this study that were randomly allocated to the program 1 cluster, were also allocated to the program 2 cluster the following year and those allocated to the control 1 cluster to the control 2 cluster. Program 2 efficacy was assessed between December 10^th^ and December 20^th^ 2006. The study authors reported that 20 clusters were randomly allocated to program 2 and 23 clusters allocated to control 2. In both clusters, approximately a quarter of HCWs were nurses (*n* = 347 in the intervention group and *n* = 339 in the control group) (Table 2). All other HCWs in this study consisted of physicians and nursing auxiliaries (Rothan-Tondeur et al., 2011).

### The intervention

There were multiple components to the intervention implemented in this study. The objective of the intervention program (referred to as program 2) was to involve HCWs in creating a “safety zone” which the influenza virus could not get through. The program included two Kits. The aim of Kit 1 was to improve HCWs vaccination uptake and Kit 2 was to reward healthcare settings when an increase in vaccination uptake was observed. Kit 1 included a slide show, posters, two booklets/leaflets, and rubber bracelets for staff. The local investigator in each of the included healthcare settings contacted a number of HCWs previously identified as opinion leaders to provide support to them and assist in promoting the influenza vaccine. Those HCWs identified as opinion leaders and whom agreed to promote the vaccine, delivered the “myths and reality about flu vaccination” slide-show to staff and answered questions. Posters indicating that the department was combating influenza were also displayed on the doors within each healthcare setting. One information leaflet was disseminated by the HCWs to families visiting their elderly residents and the other was kept for the HCWs. A rubber bracelet with the message “all together against flu” was given to all vaccinated HCWs. When HCWs flu vaccination reached >50% uptake, the healthcare setting received Kit 2 which comprised of posters indicating that the department had reached its objective. These posters were displayed on the department doors and seen by the HCWs, the elderly residents and their families (p128).

### Primary outcome

Vaccination uptake rates of nurses increased significantly in the intervention group compared to the control group (49% vs 27%, *p* < .01) (Table 2). A change in nurses’ baseline vaccination uptake rates was observed in the intervention and control group; 33% to 49% and 36% to 27%, respectively. This observation was assessed over one influenza season.

## Discussion

The main objective of this review was to provide a synthesis of the evidence which assessed the effectiveness of interventions on the seasonal influenza vaccination uptake among nurses. The EPOC taxonomy of health systems interventions informed this review to ensure that methodologically rigorous study designs were included. Only one study met the inclusion criteria (Rothan-Tondeur et al., 2011) and evaluated the effectiveness of a multiple component intervention program on the seasonal influenza vaccination uptake by occupational category. Although the included study concluded that the intervention program was effective in increasing the seasonal influenza vaccination among all occupational groups of HCWs; non-vaccinated nurses were particularly receptive to the intervention program (Rothan-Tondeur et al., 2011). Although the vaccination uptake rate in this study remained low, a change in nurses’ baseline vaccination uptake rates was observed in the intervention and control groups; 33% to 49% and 36% to 27%, respectively. The results also highlighted that the influenza vaccination uptake varied among the different occupational health groups with physicians more likely to accept the vaccine compared to nurses (91% vs 49% in the intervention group and 63% vs 27% in the control group) (Rothan-Tondeur et al., 2011). Despite the improvement observed in the vaccine uptake rate of nurses in this study, the vaccine uptake remained considerably low. Multiple component or multifaceted interventions have been reported in a number of systematic reviews to be more successful compared to single components in achieving higher vaccination uptake rates among HCWs (Bechini et al., 2020; Hollmeyer et al., 2013; Lam et al., 2010; Lytras et al., 2016; Rashid et al., 2016). However, these systematic reviews did not provide a breakdown of the effectiveness of these interventions by category of HCWs, for example, nurses. However, evaluating multifaceted interventions can make the effect of specific interventions difficult to establish. The randomised trial included in this review was conducted over a single influenza season and the short duration evaluating the intervention may impact the accuracy of the actual effect observed. The impact of interventions are encouraged to be observed over time, to improve the accuracy of the observed outcomes (Lam et al., 2010).

In comparison to multiple component interventions and although mandatory vaccination programmes have not been evaluated through methodologically rigorous study designs (Rashid et al., 2016), previous systematic reviews have reported mandatory vaccination to be the most relevant single intervention effective at increasing vaccination uptake rates of HCWs (Hollmeyer et al., 2013; Rashid et al., 2016). In the Unites States (US), HCWs’ vaccination coverage is reported to be highest among personnel who are required by their employers to be vaccinated and lowest among HCWs employed in settings where vaccination is not required, promoted or offered on site (Black et al., 2018). Although several studies have showed support for this policy (Hollmeyer et al., 2013), the concept of implementing mandatory vaccination raises many ethical issues especially among nurses (Stead et al., 2019). Making vaccination a mandatory condition of initial and continued employment is likely to be the most controversial, yet it is possibly the most likely successful method for increasing uptake (Bechini et al., 2020; Hollmeyer et al., 2013; Lytras et al., 2016). Despite this, a recent systematic review suggested better results in HCWs vaccination uptake were in fact obtained by combining interventions in different areas (Bechini et al., 2020).

Based on the results of our systematic review, the randomised trial included in this systematic review did report a significant increase in the vaccination uptake among nurses as a result of an intervention program with multiple components over a single influenza season. Program 1 of this study implemented a single component intervention which aimed to provide top down scientific information and develop a sense of altruism to the same population and healthcare settings in France (Rothan-Tondeur et al., 2009). In comparison to program 2, program 1 was ineffective in promoting vaccination uptake rates of HCWs. Although there was no baseline vaccination data provided for nurses before the implementation of program 1, the authors reported that there was no statistically significant difference observed between the two clusters in the percentage of vaccinated nurses and also nursing auxiliaries (*p* > .05). However, this program (program 1) was found to be effective for physicians and other HCWs (*p* < .05) (Rothan-Tondeur et al., 2009). This finding suggests that although multiple components may be more effective for nurses, the content of program 1 (top down scientific evidence and a sense of altruism) was met with resistance among nurses.

The findings from this review recommend high quality research studies are conducted and studies that aim to evaluate influenza campaigns or interventions should have a comparable control group. The evaluation of the intervention programmes should be provided by each occupational category and not by all HCWs in general as it is important to identify the effectiveness of interventions by the different categories of HCWs. Evidence suggests that the reasons HCWs accept or reject the seasonal influenza vaccine differ, therefore supporting the need to develop and tailor interventions differently when targeting each category of HCWs (Bouadma et al., 2012). The evidence in this systematic review on interventions aimed to increase the seasonal influenza vaccination among nurses is very limited.

The main strength of this review is found in the comprehensive search and the independent study selection, risk of bias assessment and data extraction procedures. This review followed a detailed protocol (Flanagan et al., 2020) and adhered to the selection criteria recommended in the EPOC taxonomy of health systems interventions. However, there are some limitations associated with this systematic review. Despite a comprehensive search strategy, some studies might not have been included. Many other study designs were excluded as they did not meet our pre-specified inclusion criteria. Studies that failed to report the outcome measure (i.e. vaccine uptake rate for nurses) or baseline vaccination rate for nurses before the study began were also excluded. Moreover, this review did not include studies assessing the pandemic vaccine. Therefore, this rigorous approach resulted in only one study eligible for inclusion. In addition, the risk of bias assessment indicated that there was a risk of bias present in the included study. It was also not possible to conduct a meta-analysis using Revman (RevMan) and also to assess statistical heterogeneity (Higgins and Green, 2011) as the data did not permit carrying out this analysis.

Detailed results from excluded studies are also not provided, however, studies that have previously explored this topic have done so within the context of all HCWs and therefore it was not possible to distinguish the true effect of the intervention on vaccine uptake rates by individual occupational groups.

## Conclusions

Vaccination is a complex behaviour. Although vaccination uptake rates of HCWs vary, vaccinating HCWs against seasonal influenza has been shown to be effective in protecting service users, patients and staff. Despite this, vaccination uptake amongst HCWs especially among nurses remains suboptimal. A limited number of controlled studies were available on interventions aimed at improving nurses’ vaccination rate and found that most of the literature identified uncontrolled studies of multifaceted campaigns among HCWs in general. This systematic review revealed a gap in the evidence about the appropriate interventions for increasing influenza vaccination among nurses. In view of the likelihood of influenza and the coronavirus (COVID-19) pandemic occurring together, further high quality research studies are urgently needed to assess the impact of interventions aimed at increasing the seasonal influenza vaccination uptake among nurses.

## Supplemental Material

Supplemental Material - The effectiveness of interventions to improve the seasonal influenza vaccination uptake among nurses: A systematic review Click here for additional data file. Supplemental Material for The effectiveness of interventions to improve the seasonal influenza vaccination uptake among nurses: A systematic review by Paula Catherine Flanagan, Maura Dowling, Duygu Sezgin, Jolita Mereckiene, Louise Murphy, Martina Giltenane, Peter Carr and Georgina Gethin in Journal of Infection Prevention
