# Supplementary material for: The effectiveness of interventions to improve the seasonal influenza vaccination uptake among nurses: A systematic review
Source: J Infect Prev. 2023 Oct 20;24(6):268–77. doi: 10.1177/17571774231208115 (PMC10638950; doi:10.1177/17571774231208115)
Supplement: Supplemental Material - The effectiveness of interventions to improve the seasonal influenza vaccination uptake among nurses: A systematic review [file sj-pdf-1-bji-10.1177_17571774231208115.pdf]

Supplementary material. Search strategy, Medline database

1	exp Influenza, Human/	47562
2	influenza.tw.	82645
3	seasonal influenza.tw.	3837
4	flu.tw.	10609
5	1 or 2 or 3 or 4	96551
6	exp Vaccination/	82087
7	exp Immunization/	170806
8	exp Vaccines/	222919
9	vaccination.tw.	110870
10	immunisation.tw.	8623
11	immunization.tw.	83784
12	vaccine*.tw.	199267
13	6 or 7 or 8 or 9 or 10 or 11 or 12	404479
14	5 and 13	32092
15	exp Influenza Vaccines/	22107
16	influenza vaccin*.tw.	14901
17	seasonal influenza vaccin*.tw.	1564
18	flu vaccin*.tw.	1025
19	influenza immunisation.tw.	145
20	influenza immunization.tw.	986
21	15 or 16 or 17 or 18 or 20	25186
22	14 or 21	33271
23	exp Health Personnel/	496168
24	health care personnel.tw.	2120
25	healthcare personnel.tw.	1205
26	healthcare workers.tw.	6757

27	health care workers.tw.	10002
28	exp Nurses/	86391
29	nurse*.tw.	237189
30	exp Nursing Staff/	65043
31	nursing staff.tw.	10291
32	nursing personnel.tw.	2457
33	23 or 24 or 25 or 26 or 27 or 28 or 29 or 30 or 31 or 32	657488
34	22 and 33	2582

Supplementary table 1. List of excluded studies (n=133)

**Author**	**Title**	**Reason for exclusion**
Septimus et al (2011)	A multifaceted mandatory patient safety program and seasonal influenza vaccination of health care workers in community hospitals	Wrong study design. Not a randomised trial, non-randomised trial, controlled before and after study, interrupted time series or repeated measure study
Dunais et al (2006)	Influenza vaccination: impact of an intervention campaign targeting hospital staff	Commentary only
Slaunwhite et al (2009)	Increasing vaccination rates among health care workers using unit "champions" as a motivator	Outcome data not provided by nurses only
Fujita et al (2009)	Trial of influenza HA vaccination for healthcare workers in consecutive years	Wrong intervention
Tao et al (2010)	Description of an influenza vaccination campaign and use of a randomized survey to determine participation rates	Outcome data not provided by nurses only

Sand et al (2007)	Increasing influenza immunization for long-term care facility staff using quality improvement	Not an intervention study
Giordano (2007)	Coming of age. RTs can encourage flu vaccinations	Not in English
Chouksey (2013)	Impact of mandatory influenza vaccination initiative on a major teaching hospital	Wrong population
Riphagen-Dalhuisen et al (2013)	Planning and process evaluation of a multi-faceted influenza vaccination implementation strategy for health care workers in acute health care settings	Not an intervention study
Borgey et al (2019)	Effectiveness of an intervention campaign on influenza vaccination of professionals in nursing homes: A cluster-randomized controlled trial	Outcome data not provided by nurses only
Lee and Fong (2007)	On-site influenza vaccination arrangements improved influenza vaccination rate of employees of a tertiary hospital in Singapore	Wrong study design. Not a randomised trial, non-randomised trial, controlled before and after study, interrupted time series or repeated measure study
Jegede et al (2012)	Implementing mandatory influenza vaccination policy for health care workers at a long-term acute care facility	Wrong study design. Not a randomised trial, non-randomised trial, controlled before and after study, interrupted time series or repeated measure study
Abraham et al (2013)	Improving employee flu vaccinations: The impact of leadership support	Wrong study design. Not a randomised trial, non-randomised trial, controlled before and after study, interrupted time series or repeated measure study
McCullers et al (2006)	Increased influenza vaccination of healthcare workers at a pediatric cancer hospital: results of a comprehensive influenza vaccination campaign	Wrong study design. Not a randomised trial, non-randomised trial, controlled before and after study, interrupted time series or repeated measure study
Faris et al (2009)	A system wide approach to improving healthcare workers influenza vaccination compliance	Wrong study design. Not a randomised trial, non-randomised trial, controlled before and after study, interrupted time series or repeated measure study

Chambers et al (2015)	A new approach to improving healthcare personnel influenza immunization programs: A randomized controlled trial	Outcome data not provided by nurses only
Gjerde and Kilhus (2019)	How to achieve 80% influenza vaccination coverage in a large Norwegian university hospital	Wrong study design. Not a randomised trial, non-randomised trial, controlled before and after study, interrupted time series or repeated measure study
Bautista et al (2006)	Predisposing, reinforcing, and enabling factors influencing influenza vaccination acceptance among healthcare workers	Not an intervention study
Hitzeman and Dyer (2010)	Influenza vaccination of health care personnel working with older patients	Not an intervention study
De Wals and Drapeau (1996)	Study of immunization promotion against influenza in nursing homes and long-term care facilities in Quebec	Not in English
Squeri et al (2017)	Management of two influenza vaccination campaign in health care workers of a university hospital in the south Italy	Wrong study design. Not a randomised trial, non-randomised trial, controlled before and after study, interrupted time series or repeated measure study.
Leong et al (2013)	Improving immunization (IMM) rates among oncology patients and health care providers (HCP) through a partnership of continuing medical education (CME) and quality improvement (QI) in an NCI-designated comprehensive cancer center	Outcome data not provided by nurses only
Mustafa (2017)	Improving influenza vaccination rates of healthcare workers: A multipronged approach in Qatar	Wrong study design. Not a randomised trial, non-randomised trial, controlled before and after study, interrupted time series or repeated measure study.
Polgreen et al (2010)	Prioritizing healthcare worker vaccinations on the basis of social network analysis	Not an intervention study
Robinson et al (2016)	A regional approach to improving healthcare worker influenza vaccination uptake-the Donegal staff influenza vaccination campaign	Not an intervention study

Sartor (2004)	Use of a mobile cart influenza program for vaccination of hospital employees	Wrong study design. Not a randomised trial, non-randomised trial, controlled before and after study, interrupted time series or repeated measure study.
Apisarnthanarak (2010)	Reduction of seasonal influenza transmission among healthcare workers in an intensive care unit: A 4-year intervention study in Thailand	Wrong outcomes
Chambers et al (2010)	Randomized trial of the impact of the Ottawa influenza decision aid on healthcare personnel confidence to be immunized	Conference abstract only
Oguz (2019)	Improving influenza vaccination uptake among healthcare workers by on-site influenza vaccination campaign in a tertiary children hospital	Wrong study design. Not a randomised trial, non-randomised trial, controlled before and after study, interrupted time series or repeated measure study.
Lytras (2016)	Interventions to increase seasonal influenza vaccine coverage in healthcare workers: A systematic review and meta-regression analysis	Systematic review
Chamoux (2006)	Impact study of an active anti flu vaccination programme on the Clermont-Ferrand teaching hospital staff	Outcome data not provided by nurses only
Chamber et al (2012)	Impact of the Ottawa Influenza Decision Aid on healthcare personnel's influenza immunization decision: A randomized trial	Outcome data not provided by nurses only
Dey et al (2001)	Promoting uptake of influenza vaccination among health care workers: A randomized controlled trial	Outcome data not provided by nurses only
Garcell et al (2015)	A Successful Strategy for Improving the Influenza Immunization Rates of Health Care Workers without a Mandatory Policy	Not an intervention study
Roggendorf et al (2018)	Improvement of influenza vaccination rates among health care workers by personalised "STOP-Influenza" campaign in a university hospital	Not in English
Karanfil et al (2011)	Championing patient safety through mandatory influenza vaccination for all healthcare personnel and affiliated physicians	Outcome data not provided by nurses only

Calabria et al (2014)	Impact of patient safety and legislative standards on healthcare worker vaccination rates: A single center experience	Outcome data not provided by nurses only
Moody et al (2012)	Preventing the flu in you: A three-year experience of sustained seasonal influenza vaccination rates in healthcare workers	Wrong study design. Not a randomised trial, non-randomised trial, controlled before and after study, interrupted time series or repeated measure study.
Sawyer et al (2012)	A public health initiative to increase annual influenza immunization among hospital health care personnel: The San Diego Hospital Influenza Immunization Partnership	Wrong study design. Not a randomised trial, non-randomised trial, controlled before and after study, interrupted time series or repeated measure study.
Currie and Malow (2012)	Successful implementation of a mandatory influenza vaccination program across a 12-hospital system	Outcome data not provided by nurses only
McCormack (2011)	How to run a successful flu campaign	Not an intervention study
Llupià et al (2010)	New interventions to increase influenza vaccination rates in health care workers	Wrong study design. Not a randomised trial, non-randomised trial, controlled before and after study, interrupted time series or repeated measure study.
Cheung et al (2017)	Pattern of exposure to information and its impact on seasonal influenza vaccination uptake in nurses	Not an intervention study
Alicino et al (2015)	Influenza vaccination among healthcare workers in Italy: The experience of a large tertiary acute-care teaching hospital	Outcome data not provided by nurses only
Ksienski (2014)	Mandatory seasonal influenza vaccination or masking of British Columbia health care workers: Year 1	Wrong study design. Not a randomised trial, non-randomised trial, controlled before and after study, interrupted time series or repeated measure study.
Tognetto et al (2019)	Seasonal influenza vaccination among health-care workers: the impact of different tailored programs in four University hospitals in Rome<br><br>DOI: 10.1080/21645515.2019.1632684	Outcome data not provided by nurses only

MMWR (2005)	Interventions to increase influenza vaccination of health-care workers--California and Minnesota	Not an intervention study
Ribner et al (2008)	Use of a mandatory declination form in a program for influenza vaccination of healthcare workers	Wrong study design. Not a randomised trial, non-randomised trial, controlled before and after study, interrupted time series or repeated measure study.
Bohm et al (2016)	Exploring and Promoting Prosocial Vaccination: A Cross-Cultural Experiment on Vaccination of Health Care Personnel	Not an intervention study
Bressin et al (2019)	Evaluation of a multimodal strategy to promote influenza vaccination of healthcare workers	Wrong study design. Not a randomised trial, non-randomised trial, controlled before and after study, interrupted time series or repeated measure study.
Navalón Ramon et al (2019)	Use of mobile immunization teams to increase influenza vaccination coverage among healthcare workers. A community intervention trial	Outcome data not provided by nurses only
Freshman et al (2015)	Implementation of a "free-choice" Influenza Vaccination Policy Improves Vaccination Compliance among Healthcare Workers in a Pennsylvania Healthcare System.	Wrong study design. Not a randomised trial, non-randomised trial, controlled before and after study, interrupted time series or repeated measure study.
Costantino et al (2019)	Effectiveness of an educational intervention on seasonal influenza vaccination campaign adherence among healthcare workers of the Palermo University Hospital, Italy	Outcome data not provided by nurses only
Camargo-Ángeles et al (2014)	Evaluation of a novel flu vaccination campaign among health personnel for the 2011-2012 season	Not in English
Giordano (2000)	Coming of age. RTs can encourage flu vaccinations	Wrong study design. Not a randomised trial, non-randomised trial, controlled before and after study, interrupted time series or repeated measure study.
Esolen and Kilheeney (2014)	Sustaining high influenza vaccination compliance with a mandatory masking program	Wrong study design. Not a randomised trial, non-randomised trial, controlled before and after study, interrupted time series or repeated measure study.

Dille (1999)	Worksite influenza immunization. Successful program	Wrong study design. Not a randomised trial, non-randomised trial, controlled before and after study, interrupted time series or repeated measure study.
Nace et al (2011)	Increasing influenza and pneumococcal immunization rates in a nursing home network	Wrong study design. Not a randomised trial, non-randomised trial, controlled before and after study, interrupted time series or repeated measure study.
Iten et al (2019)	Giving healthcare workers (HCW) a choice between vaccination and wearing a mask during seasonal influenza epidemic: A possible strategy to increase vaccination coverage	Wrong study design. Not a randomised trial, non-randomised trial, controlled before and after study, interrupted time series or repeated measure study.
Rodríguez-Fernández et al (2016)	Impact of an influenza vaccine educational programme on healthcare personnel	Wrong study design. Not a randomised trial, non-randomised trial, controlled before and after study, interrupted time series or repeated measure study.
Podczervinski et al (2015)	Employee influenza vaccination in a large cancer center with high baseline compliance rates: Comparison of carrot versus stick approaches	Wrong study design. Not a randomised trial, non-randomised trial, controlled before and after study, interrupted time series or repeated measure study.
Polgreen et al (2008)	Elements of influenza vaccination programs that predict higher vaccination rates: Results of an emerging infections network survey	Not an intervention study
Quan et al (2012)	Voluntary to mandatory: Evolution of strategies and attitudes toward influenza vaccination of healthcare personnel	Not an intervention study
Sangil et al (2012)	Influenza vaccination uptake among health care workers after a multimodal educational campaign in the post pandemic year	Wrong study design. Not a randomised trial, non-randomised trial, controlled before and after study, interrupted time series or repeated measure study.
Yue et al (2017)	Workplace interventions associated with influenza vaccination coverage among health care personnel in ambulatory care settings during the 2013-2014 and 2014-2015 influenza seasons	Outcome data not provided by nurses only
Tapiainen et al (2005)	Influenza vaccination among healthcare workers in a university children's hospital	Wrong study design. Not a randomised trial, non-randomised trial, controlled before and after study, interrupted time series or repeated measure study.

Tatar et al (2016)	The achoo stops with you: How several strategies, including mandatory healthcare worker (HCW) influenza (FLU) vaccine program, led to increases in vaccine compliance and decreases in healthcare-acquired (HA) Flu	Outcome data not provided by nurses only
Bert et al (2019)	A new strategy to promote flu vaccination among health care workers: Molinette Hospital's experience	Wrong study design. Not a randomised trial, non-randomised trial, controlled before and after study, interrupted time series or repeated measure study.
Riphagen-Dalhuisen et al (2013)	Hospital-based cluster randomised controlled trial to assess effects of a multi-faceted programme on influenza vaccine coverage among hospital healthcare workers and nosocomial influenza in the Netherlands, 2009 to 2011	Outcome data not provided by nurses only
García De Codes et al (2010)	Influenza (pandemic and seasonal) vaccination campaign among workers of a general hospital (2009-2010)	Wrong study design. Not a randomised trial, non-randomised trial, controlled before and after study, interrupted time series or repeated measure study.
Friedl et al (2012)	An intensive 5-year-long influenza vaccination campaign is effective among doctors but not nurses	Wrong study design. Not a randomised trial, non-randomised trial, controlled before and after study, interrupted time series or repeated measure study.
Palmore et al (2009)	A successful mandatory influenza vaccination campaign using an innovative electronic tracking system	Wrong study design. Not a randomised trial, non-randomised trial, controlled before and after study, interrupted time series or repeated measure study.
O'Donnell et al (2012)	The stop (staff taking ownership for prevention) flu initiative: Improving influenza vaccination rates among staff in a long-term care facility	Wrong study design. Not a randomised trial, non-randomised trial, controlled before and after study, interrupted time series or repeated measure study.
Maltezou et al (2008)	Strategies to increase influenza vaccine uptake among health care workers in Greece	Wrong study design. Not a randomised trial, non-randomised trial, controlled before and after study, interrupted time series or repeated measure study.
Conte et al (2016)	Promotion of flu vaccination among healthcare workers in an Italian academic hospital: An experience with tailored web tools	Wrong study design. Not a randomised trial, non-randomised trial, controlled before and after study, interrupted time series or repeated measure study.

Bandaly et al (2009)	Impact of the vaccination recommendations against influenza on the perceptions and motivations of the professionals of an emergency-SMUR-Short stay medical unit department	Wrong study design. Not a randomised trial, non-randomised trial, controlled before and after study, interrupted time series or repeated measure study.
Looijmans-van den Akker et al (2011)	How to develop a program to increase influenza vaccine uptake among workers in health care settings?	Not an intervention study
Lavela et al (2015)	Healthcare worker influenza declination form program	Wrong study design. Not a randomised trial, non-randomised trial, controlled before and after study, interrupted time series or repeated measure study.
Gilardi et al (2018)	Seasonal Influenza Vaccination in Health Care Workers. A Pre-Post Intervention Study in an Italian Paediatric Hospital	Wrong study design. Not a randomised trial, non-randomised trial, controlled before and after study, interrupted time series or repeated measure study.
Haney (2015)	Fight the flu: Increasing staff compliance with influenza vaccination	Wrong study design. Not a randomised trial, non-randomised trial, controlled before and after study, interrupted time series or repeated measure study.
Kung (2014)	A quality improvement project to increase influenza vaccination in healthcare personnel at a university health center	Wrong study design. Not a randomised trial, non-randomised trial, controlled before and after study, interrupted time series or repeated measure study.
Tannenbaum et al (1993)	Evaluation of an influenza vaccination program for nursing home staff	Outcome data not provided by nurses only
Lehmann et al (2016)	Changing the default to promote influenza vaccination among health care workers	Outcome data not provided by nurses only
MMWR (2005)	Interventions to increase influenza vaccination of health-care workers - California and Minnesota	Wrong study design. Not a randomised trial, non-randomised trial, controlled before and after study, interrupted time series or repeated measure study.
Riphagen-Dalhuisen et al (2012)	Effects of a multi-faceted program to increase influenza vaccine coverage among health care workers: A hospital-based cluster randomized controlled trial	Conference abstract only

Booy et al (2011)	Mandating influenza vaccination in health-care workers	Not an intervention study
Marwaha et al (2016)	GET POKED: Comparing an Incentive-Based Flu Campaign with Vaccinate-or-Mask Policies to Boost Influenza Vaccination Rates Among Healthcare Workers	Outcome data not provided by nurses only
Edelstein and Pebody (2013)	Can we achieve high uptakes of influenza vaccination of healthcare workers in hospitals? A cross-sectional survey of acute NHS trusts in England	Not an intervention study
Parada et al (2013)	Four-year experience with mandatory seasonal influenza immunization for all personnel in a university medical center	Outcome data not provided by nurses only
Floyd (2013)	Mandatory influenza vaccination program proves successful in its first year	Not an intervention study
Leibu and Maslow (2015)	Effectiveness and acceptance of a health care-based mandatory vaccination program	Wrong study design. Not a randomised trial, non-randomised trial, controlled before and after study, interrupted time series or repeated measure study.
Jung et al (2017)	Stepwise intervention including 1-on-1 counselling is highly effective in increasing influenza vaccination among health care workers	Wrong study design. Not a randomised trial, non-randomised trial, controlled before and after study, interrupted time series or repeated measure study.
Elawad et al (2017)	Improving influenza vaccination rate among primary healthcare workers in Qatar	Wrong study design. Not a randomised trial, non-randomised trial, controlled before and after study, interrupted time series or repeated measure study.
Cassidy (2001)	Flu campaign targets healthcare workers	Wrong study design. Not a randomised trial, non-randomised trial, controlled before and after study, interrupted time series or repeated measure study.
Heinrich-Morrison et al (2015)	An effective strategy for influenza vaccination of healthcare workers in Australia: Experience at a large health service without a mandatory policy	Wrong study design. Not a randomised trial, non-randomised trial, controlled before and after study, interrupted time series or repeated measure study.

Vimercati et al (2019)	Influenza vaccination in health-care workers: an evaluation of an on-site vaccination strategy to increase vaccination uptake in HCWs of a South Italy Hospital	Wrong study design. Not a randomised trial, non-randomised trial, controlled before and after study, interrupted time series or repeated measure study.
McCarty et al (2010)	Helping healthcare workers decide: evaluation of an influenza immunization decision tool	Not an intervention study
McNally et al (2018)	Influenza vaccination uptake in healthcare workers in Ireland: Effectiveness of a brief educational intervention in promoting positive attitudinal change	Wrong study design. Not a randomised trial, non-randomised trial, controlled before and after study, interrupted time series or repeated measure study.
Edwards et al (2016)	Impact of the flu mask regulation on health care personnel influenza vaccine acceptance rates	Not an intervention study
Parada et al (2011)	No more Mr Nice Guy - Implementation of mandatory seasonal influenza immunization for all personnel	Wrong study design. Not a randomised trial, non-randomised trial, controlled before and after study, interrupted time series or repeated measure study.
Walsh et al (2012)	The FLU-FOBT Program in community clinics: durable benefits of a randomized controlled trial	Wrong intervention
Butteri et al (2010)	Flu in 15: A novel 15-minute education program to promote acceptance of the influenza vaccine among health care workers	Wrong study design. Not a randomised trial, non-randomised trial, controlled before and after study, interrupted time series or repeated measure study.
Gaughan (2010)	The successful implementation of mandatory seasonal influenza vaccination for health care workers at an academic medical center	Wrong study design. Not a randomised trial, non-randomised trial, controlled before and after study, interrupted time series or repeated measure study.
Batabyal et al (2017)	Impact of New York State Influenza Mandate on Influenza-Like Illness, Acute Respiratory Illness, and Confirmed Influenza in Healthcare Personnel	Wrong study design. Not a randomised trial, non-randomised trial, controlled before and after study, interrupted time series or repeated measure study.
North et al (2013)	A novel two-tiered approach to increase healthcare worker influenza vaccination rates	Wrong study design. Not a randomised trial, non-randomised trial, controlled before and after study, interrupted time series or repeated measure study.

Llupia et al (2013)	Evaluating influenza vaccination campaigns beyond coverage: A before-after study among health care workers	Wrong study design. Not a randomised trial, non-randomised trial, controlled before and after study, interrupted time series or repeated measure study.
Cadena et al (2011)	Improving influenza vaccination of healthcare workers by means of quality improvement tools	Outcome data not provided by nurses only
Rakita et al (2010)	Mandatory influenza vaccination of healthcare workers: A 5-year study	Outcome data not provided by nurses only
Braxton (2010)	Do multiple interventions improve influenza vaccination compliance rates among nursing staff at the Hampton Veterans Administration Medical Center?	Wrong study design. Not a randomised trial, non-randomised trial, controlled before and after study, interrupted time series or repeated measure study.
Pan et al (2015)	Increased flu vaccination rate due to utilize of mobile cart of vaccination	Wrong study design. Not a randomised trial, non-randomised trial, controlled before and after study, interrupted time series or repeated measure study.
Kamali (2018)	Increasing healthcare personnel influenza vaccination coverage in Los Angeles County hospitals	Wrong study design. Not a randomised trial, non-randomised trial, controlled before and after study, interrupted time series or repeated measure study.
Albany et al (2009)	Success of flu vaccine for employees: Collaborative approach to increasing the number of HCW participation in flu campaign	Wrong study design. Not a randomised trial, non-randomised trial, controlled before and after study, interrupted time series or repeated measure study.
Faris et al (2012)	First do no harm-efficacy of influenza vaccine mandate or mask mandate for the healthcare worker	Wrong study design. Not a randomised trial, non-randomised trial, controlled before and after study, interrupted time series or repeated measure study.
Huynh et al (2012)	Mandatory influenza vaccination of health care workers: A first-year success implementation by a community health care system	Wrong study design. Not a randomised trial, non-randomised trial, controlled before and after study, interrupted time series or repeated measure study.
Honda et al (2013)	A multifaceted intervention to increase the influenza vaccination rate of healthcare workers without a mandatory policy in Japan	Wrong study design. Not a randomised trial, non-randomised trial, controlled before and after study, interrupted time series or repeated measure study.

Doratotaj et al (2008)	A novel approach to improve influenza vaccination rates among health care professionals: A prospective randomized controlled trial	Outcome data not provided by nurses only
Ffrench and Ellison (1970)	An influenza vaccination programme for hospital staff winter 1968-1969	Wrong outcomes
Peterson et al (2015)	The power of involving house staff in quality improvement: an interdisciplinary house staff-driven vaccination initiative	Wrong study design. Not a randomised trial, non-randomised trial, controlled before and after study, interrupted time series or repeated measure study.
Perlin et al (2013)	Developing a program to increase seasonal influenza vaccination of healthcare workers: lessons from a system of community hospitals	Wrong study design. Not a randomised trial, non-randomised trial, controlled before and after study, interrupted time series or repeated measure study.
Lopes et al (2008)	Intervention to increase influenza vaccination rates among healthcare workers in a tertiary teaching hospital in Brazil	Wrong study design. Not a randomised trial, non-randomised trial, controlled before and after study, interrupted time series or repeated measure study.
Kim et al (2015)	Evaluation of the impact of the 2012 Rhode Island health care worker influenza vaccination regulations: implementation process and vaccination coverage	Not an intervention study
De Juanes et al (2007)	Influenza vaccination coverage among hospital personnel over three consecutive vaccination campaigns (2001-2002 to 2003-2004)	Wrong study design. Not a randomised trial, non-randomised trial, controlled before and after study, interrupted time series or repeated measure study.
Hitoshi et al (2013)	A Successful Strategy for Increasing the Influenza Vaccination Rate of Healthcare Workers without a Mandatory Policy Outside of the United States: A Multifaceted Intervention in a Japanese Tertiary Care Center	Wrong study design. Not a randomised trial, non-randomised trial, controlled before and after study, interrupted time series or repeated measure study.
Hirsch et al (2011)	Seasonal influenza vaccination of healthcare employees: Results of a 4-year campaign	Outcome data not provided by nurses only
Drees et al (2015)	Carrots and Sticks: Achieving High Healthcare Personnel Influenza Vaccination Rates without a Mandate	Outcome data not provided by nurses only

Thomas (2010)	A promotional, educational intervention increased staff influenza vaccination in primary care	Commentary only
Venci et al (2015)	Inclusion of social media-based strategies in a health care worker influenza immunization campaign	Commentary only
Song et al (2006)	Effect of a hospital campaign for influenza vaccination of healthcare workers	Wrong study design. Not a randomised trial, non-randomised trial, controlled before and after study, interrupted time series or repeated measure study.
McFadden and Sadler (2011)	Implementation of a mandatory influenza vaccination program in an academic medical center	Wrong study design. Not a randomised trial, non-randomised trial, controlled before and after study, interrupted time series or repeated measure study.
Looijmans-Van Den Akker et al (2010)	Effects of a multi-faceted program to increase influenza vaccine uptake among health care workers in nursing homes: A cluster randomised controlled trial	Baseline vaccine uptake rates not reported for nurses
Abramson et al (2010)	Randomized Trial of a Program to Increase Staff Influenza Vaccination in Primary Care Clinics	Baseline vaccine uptake rates not reported for nurses
Rothan-Tondeur et al (2009)	Assessment of healthcare worker influenza vaccination program in French geriatric wards: A cluster-randomized controlled trial	Baseline vaccine uptake rates not reported for nurses
Harbarth et al (1998)	Influenza immunization: improving compliance of healthcare workers	Baseline vaccine uptake rates not reported for nurses
